# Prognostic value of preoperative neutrophil-to-lymphocyte ratio in Crohn’s disease

**DOI:** 10.1007/s00508-018-1322-3

**Published:** 2018-02-12

**Authors:** Stanislaus Argeny, Anton Stift, Michael Bergmann, Martina Mittlböck, Svenja Maschke, Yushan Yang, Praminthra Chitsabesan, Stefan Riss

**Affiliations:** 1 Department of Surgery, Medical University of Vienna, Währinger Gürtel 18–20, 1090 Vienna, Austria; 2 Center for Medical Statistics, Informatics, and Intelligent Systems, Medical University of Vienna, 1090 Vienna, Austria; 3 NHS Foundation Trust, York Teaching Hospital, YO31 8HE York, UK

**Keywords:** Crohn’s disease, Neutrophil-to-lymphocyte ratio, Complication, Cancer, Surgery

## Abstract

**Background:**

The Neutrophil-to-lymphocyte-ratio has recently gained increased attention as a prognostic marker for malignant disease and short term outcomes. There is little data available in patients with Crohn’s disease, thus the present study was conducted to correlate preoperative Neutrophil-to-lymphocyte-ratio values with disease phenotype and postoperative course.

**Methods:**

We comprised 373 patients, who underwent intestinal resection for symptomatic Crohn’s disease at an academic tertiary referral centre between 2000 and 2014. Preoperative Neutrophil-to-lymphocyte-ratio values were calculated and analyzed in regard to disease phenotype and 30-day morbidity rate. All relevant data were obtained from the institutional database and individual chart review.

**Results:**

Male patients had significantly higher preoperative Neutrophil-to-lymphocyte-ratio values compared to female patients (5 vs. 4; *p* = 0.0075). A higher Neutrophil-to-lymphocyte-ratio was also found in patients with an acute indication for surgery (6.15 vs. 4.3; *p* = 0.0374), presenting with abscesses (5.36 vs. 4.28; *p* = 0.0254), inflammatory masses (5.23 vs. 4.08; *p* = 0.0294) or malignancy in the resected specimen (9.06 vs. 4.35, *p* = 0.0231). Surprisingly, patients developing postsurgical complications showed significantly lower Neutrophil-to-lymphocyte-ratio values (3.77 vs. 4.67; *p* = 0.0461).

**Conclusions:**

Elevated preoperative Neutrophil-to-lymphocyte-ratio in symptomatic Crohn’s disease is not predictive for complications. However, Neutrophil-to-lymphocyte-ratio showed a significant correlation with specific disease phenotypes. Most strikingly, Neutrophil-to-lymphocyte-ratio was highly elevated in patients with a colorectal cancer in the resected specimen, which needs to be addressed in future studies.

## Introduction

Crohn’s disease (CD) is a chronic disease with fluctuating mucosal inflammation, which can affect the entire gastrointestinal tract. Relapses are common and surgery still remains a cornerstone in the overall treatment strategy. The ability to predict the course of disease or the phenotype in CD patients is therefore of importance for physicians and surgeons [1].

The most widely acknowledged inflammation markers for monitoring chronic and acute disease activity in inflammatory bowel disease (IBD) are C‑reactive protein (CRP), an acute phase protein with a short half-life and total white blood cell count. Both are inexpensive and can be easily used as a screening tool. In addition, various other markers, such as fecal levels of lactoferrin, calprotectin, polymorphonuclear neutrophil elastase or erythrocyte sedimentation rate, are applicable for surveying disease activity in CD patients [2, 3]. Even though CRP levels correlate with disease activity, the lack of sensitivity of CRP levels alone in initial diagnoses and measuring disease extent limits its clinical use [2–6].

In recent years, the neutrophil-to-lymphocyte ratio (NLR), calculated as total neutrophil count divided by total lymphocyte count, has gained increased attention for assessing the grade of inflammation. The NLR was initially described as a general immunological response to different stress stimuli, which correlated with outcomes and organic dysfunction scores of critically ill intensive care unit patients [7]. Notably, NLR represents two different immune pathways and can be easily derived directly from a standard blood test. Several studies found that NLR alone or in combination with other blood markers to not only predict patient survival for various malignant disease, including colorectal cancer [8–16], and survival after cardiac interventions [17], but also to predict surgical complications after major abdominal [18–20] and orthopedic operations [21]. Recent reports revealed the predictive value of NLR in discriminating active from inactive ulcerative colitis (UC), proclaiming cut-offs between 2.16 and 2.47, compared to the mixed results regarding the diagnostic value of CRP alone [22–24].

Only limited experience concerning NLR is available for CD [25, 26] and to the best of our knowledge there are no studies that address the predictive role of NLR on disease phenotype and postoperative complications. Therefore, the present investigation was designed to assess the association of NLR and short-term outcome following intestinal resections for CD.

## Patients, materials and methods

This was a retrospective study of 373 patients who underwent intestinal resection due to symptomatic CD at a tertiary referral centre between 2000 and 2014. The study was approved by the ethics committee of the Medical University Vienna.

The NLR derived from peripheral blood samples was calculated as total neutrophil count divided by total lymphocyte count. Patients who had no differential blood test available within the 14 days prior to surgery were excluded from further analysis. In addition, total leucocyte counts, CRP (mg/dl) and total thrombocytes count (G/l) were recorded from available blood test.

All operations were conducted or supervised by a single colorectal team specializing in the treatment of IBD. The laparoscopic approach has already been described previously in detail [27]. Demographic and clinical baseline data including information about surgical characteristics, laboratory values and the use of immunosuppressive medications were obtained from the institutional database and individual chart review respectively. Steroid therapy was defined as the use of corticosteroids until the day before surgery. Those patients who received steroids preoperatively but stopped treatment at least 2 days before surgery were regarded as “non-steroids” as defined in a previous study [28]. The exposure to azathioprin/6-mercaptopurin (AZA/6MP) preoperatively was defined as the use of AZA/6MP within 14 days before operation. The administration of anti-tumor necrosis factor (TNF) antibody was recorded within 7 days before surgery. Intestinal resections were divided into simple (1 intestinal resection) and complex (>1 intestinal resection), the 30-day morbidity following surgery was defined as major and minor complications and the Clavien-Dindo classification [29] recorded.

### Statistical analysis

Continuous data are shown as median, minimum and maximum due to skew distributions. Differences between groups are tested by Wilcoxon rank sum tests. Monotone trends are assessed and tested by Spearman’s rank correlation (r_s_). Categorical variables are described with absolute numbers and percentages. In the case of binary variables monotone trends are tested by a trend version of the χ^2^-test. In case of sparse data an exact χ^2^-test was calculated. All *p*-values were two-sided and *p* ≤ 0.05 was considered statistically significant. All calculations were performed with SAS version 9.3 (SAS Institute, Cary, NC, USA).

## Results

Altogether 373 (71%) patients fulfilled the study requirement of valid NLR values. Their demographic data are listed in Table 1.

**Table 1:** Demographic characteristics of patients operated on for Crohn’s disease in correlation with heutrophil-to-lymphocyte ratio (NLR)

Demographic characteristics	Median (min–max)		NLR median (min–max)	*p*-value
Body mass index (BMI)	21.09 (12.22–40.61)		4.44 (0.02–45)	0.1209
Age (years, range)	35.42 (15.42–82.40)		4.48 (0.02–45)	*0.0187*
Demographic characteristics	*N (%)*		*NLR* *median (min–max)*	*p-value*
Sex	Female	170 (45.58)	4 (0.16–30.39)	*0.0075*
	Male	203 (54.42)	5 (0.02–45)	
Corticosteroids	Yes	65 (17.43)	5.89 (0.16–45)	*0.0036*
	No	308 (82.57)	4.26 (0.02–30.38)	
Anti-TNF antibody	Yes	6 (1.61)	5.28 (2.65–10.33)	0.6660
	No	363 (98.39)	4.38 (0.02–45)	
Azathioprine/6-mercaptopurine	Yes	87 (23.32)	6.08 (1.15–24)	*0.0110*
	No	286 (76.68)	4.08 (0.02–45)	
Budesonide	Yes	46 (12.33)	5.16 (2.23–44.4)	0.5521
	No	327 (87.67)	4.38 (0.02–45)	

Data are described as *N* (%) or median (minimum–maximum).

*NLR* Neutrophil-to-lymphocyte ratio

The NLR was significantly lower in female than in male patients (*p* = 0.0075). Increasing age significantly correlated with lower NLR values (r_s_ = −0.1217; *p* = 0.0187) while higher NLR values were found in patients with AZA/6MP (*p* = 0.011) and corticosteroids (*p* = 0.0036) usage prior to surgery. Patients with the intake of anti-TNF antibodies had higher NLR values but due to the small number of patients with anti-TNF antibodies (*n* = 6) no significant differences can be shown.

### NLR and surgical characteristics

The NLR values showed significant differences in relation to disease phenotypes as displayed in Table 2. The median NLRs of patients presenting with inflammatory masses or abscesses were significantly higher than in patients without (*p* = 0.0294 and *p* = 0.0254, respectively). Additionally, an acute indication for surgery was associated with a higher ratio in contrast to elective operations (*p* = 0.0374). The type of resection (simple versus complex intestinal resection) did not reveal any difference concerning the NLR.

**Table 2:** Neutrophil-to-lymphocyte ratio (NLR) with respect to surgical characteristics in patients operated on for Crohn’s disease

Surgical characteristics		*N* (%)	NLR median (min–max)	*p*-value
Stenosis	Yes	280 (75.07)	4.32 (0.02–45)	0.1337
	No	93 (24.93)	5.2 (0.16–44.4)	
Fistula	Yes	186 (49.87)	4.97 (0.02–45)	0.1526
	No	187 (50.13)	4.33 (0.16–20)	
Inflammatory mass	Yes	156 (41.82)	5.36 (0.16–30)	*0.0294*
	No	217 (58.18)	4.28 (0.02–45)	
Abscess	Yes	89 (23.86)	5.36 (0.16–30)	*0.0254*
	No	284 (76.14)	4.28 (0.02–45)	
Malignancy	Yes	8 (2.14)	9.06 (3.7143–15.444)	*0.0231*
	No	365 (97.86)	4.35 (0.0209–45)	
Indication for surgery	Acute	37 (9.92)	6.15 (0.16–29.14)	*0.0374*
	Elective	336 (90.08)	4.3 (0.02–45)	
Type of resection	Simple (1 resection)	303 (81.23)	4.46 (0.02–45)	0.6743
	Complex (>1 resection)	70 (18.77)	4.25 (1.20–15.57)	

Data are described as *N* (%) or median (minimum–maximum).

*NLR* Neutrophil-to-lymphocyte ratio

### NLR and postoperative complications

There were a total of 82 (22%) postoperative complications. Minor complications, defined as Clavien-Dindo Grade I+II, were observed in 57 (15.3%) patients, whereas major complications (Clavien-Dindo Grade III–V) were found in 25 (6.7%) patients. Complications are described in Table 3 in more detail.

**Table 3:** Postoperative complications of 373 patients operated on for Crohn’s disease according to the Clavien-Dindo classification

Clavien-Dindo classification	Complications	*N* (%)
Grade I: *n* = 29	Wound infections	5 (1.34)
	Paralytic ileus	22 (5.90)
	Others	2 (0.54)
Grade II: *n* = 28	Wound infection	4 (1.07)
	Urinary tract infection	3 (0.80)
	Fever of unknown origin	14 (3.76)
	Pneumonia	3 (0.80)
	Others	4 (1.07)
Grade III: *n* = 20	Abscess	3 (0.80)
	Mechanical ileus	3 (0.80)
	Wound infection	4 (1.07)
	Anastomotic dehiscence	9 (2.41)
	Stoma necrosis and bleeding	1 (0.27)
Grade IV: *n* = 3	Pulmonary embolism	2 (0.54)
	Respiratory insufficiency	1 (0.27)
Grade V: *n* = 2	Multiple organ failure	1 (0.27)
	Circulatory failure	1 (0.27)

Surprisingly, NLR values were decreased in patients with postoperative complications and also depended on the severity of complications (r_s_ = 0.1041; *p* = 0.0446) (Table 4).

**Table 4:** Neutrophil-to-lymphocyte ratio (NLR) in correlation with complications after surgery for Crohn’s disease according to the Clavien-Dindo classification

Clavien-Dindo classification	*N* (%)	NLR median (min–max)	*p*-value
Grade: 0	291 (78.02)	4.67 (0.02–45)	**0.0446**
Grade: I+II	57 (15.28)	3.85 (1.15–15.44)	
Grade: III–V	25 (6.70)	3.42 (2.0–44.4)	

Data are described as *N* (%) or median (minimum–maximum).

*NLR* Neutrophil-to-lymphocyte ratio

In 296 patients a complete preoperative blood test including CRP, leucocytes and thrombocytes were recorded. The median CRP value was 1.47 mg/dl (0.02–55.5), the median total thrombocytes count was 337 G/l (117–738) and median total leucocytes count was 7.65 G/l (3.25–24.05). Notably, only CRP showed a significant negative correlation with an eventful postoperative course, whereas no association between thrombocytes, leucocytes and complications could be detected. Further details are outlined in Table 5.

**Table 5:** Neutrophil-to-lymphocyte ratio (NLR), CRP, leucocytes and thrombocytes in correlation to complications after surgery for Crohn’s disease according to the Clavien-Dindo classification

Clavien-Dindo classification	*N* (%)	CRP (mg/dl) median (min–max)	Leucocytes (G/l) median (min–max)	NLR median (min–max)	Thrombocytes (G/l) median (min–max)
Grade: 0	223 (75.34)	1.52 (0.04–55.5)	7.69 (3.55–24.04)	5.59 (0.69–45)	335 (117–738)
Grade: I+II	51 (17.23)	1.34 (0.02–10.87)	7.78 (3.25–18.28)	4 (1.15–15.44)	370 (208–782)
Grade: III–V	22 (7.43)	1.03 (0.07–24.81)	7.44 (4.67–16.05)	3.7778 (2.0–44.4)	287 (122–540)
*p-value*	–	*0.048*	0.418	0.79	0.097

Data are described as *N* (%) or median (minimum–maximum).

*CRP* C‑reactive protein, *NLR* Neutrophil-to-lymphocyte ratio

### NLR and malignant disease

Histopathological examinations of resected specimens revealed intestinal carcinomas in eight patients (Table 2). Preoperative NLR values of these patients (median 9.06) were twice as high than in patients without carcinomas (median 4.34) and was statistically significantly different (*p* = 0.0231).

## Discussion

In this retrospective study we aimed to correlate the NLR in patients operated on for symptomatic CD with preoperative factors and early postoperative complications. We revealed higher NLRs in patients presenting intraoperatively with abscesses and inflammatory masses, most likely due to severe inflammation and consequently a stronger immune system response. These patients are more likely to be on active treatment, hence why the NLR would be higher in patients with AZA/6MP and corticosteroids usage prior to surgery.

Surprisingly, an uneventful postoperative course was significantly associated with a higher NLR, although median values did not show large differences. Another highly significant finding with potential clinical relevance was that higher NLRs increased the likelihood of colorectal cancer in resected specimens. The role of systemic inflammation and its influence on higher counts of circulatory neutrophil cells and decreased counts of lymphocytes was already described by Schneider et al. [30]. Consequently, the ratio of neutrophil cells and lymphocytes has gained increased attention in the literature as it is a simple index reflecting inflammatory (neutrophils) and regulatory processes (lymphocytes) of the immune system; however, because NLR displays an overall inflammation state, its applicability to IBD and its clinical use is still under debate.

Acarturk et al. assessed NLR and CRP levels using the Harvey-Bradshaw index and the Truelove and Witts criteria, by matching 22 CD and 44 UC patients with active and inactive disease with 41 healthy controls [26]. The authors found that the mean NLR value was 5.25 in CD patients with active disease in contrast to 1.68 in the healthy control group and 1.78 in patients with inactive CD. Different results were published by Gao et al. who also compared NLR values of CD patients with active and inactive disease with those of a healthy control group. A mean NLR value of 5.72 was observed in 110 CD patients, which was not significantly different between active and inactive CD; however, NLR showed to be more predictive than CRP or total white blood cell count for discriminating CD from non-CD patients [25]. These finding might partially explain our results by showing a generally altered immune system response in CD patients compared to non-CD patients, reflected by elevated NLR and CRP values in both active as well as inactive CD. This consequently questions the applicability of NLR and its established predictive preoperative value for adverse postoperative events in non-chronic inflammation patients, to CD patients with higher overall inflammation markers.

Few studies examined the value of NLR in monitoring disease activity in UC patients. Study designs varied but median NLR in active UC ranged between 2.59 and 3.22. The NLR cut-off values between 2.16 and 2.47 were proclaimed to best discriminate active from inactive disease [22–24, 31]. Posul et al. showed in 49 UC patients that active disease was significantly associated with lower platelets and higher total white blood cell counts [22].

There is growing evidence of the predictive role of NLR as prognostic marker in malignant disease [8–16, 32]. In a meta-analysis, Li et al. demonstrated the prognostic value of elevated NLR in colorectal cancer patients leading to poorer overall survival and poorer tumor differentiation [12]. In our study cohort, eight patients had histologically proven colorectal malignancy. These patients presented preoperatively with significant higher NLRs, suggesting a correlation of NLR and malignant disease.

To the authors knowledge there exists no study that assessed the impact of NLR on short-term outcome and on disease phenotype in CD. In the line with our results, Forget et al. included 82 patients with a median age of 62 years undergoing major abdominal surgery, and found preoperative NLR values were not predictive of an eventful or uneventful postoperative course [18]. Notably, this group of patients were significantly older and not restricted to CD. In contrast, Vaughan-Shaw et al. showed a preoperatively elevated NLR to be predictive for postoperative complications in 88 elderly patients after emergency abdominal surgery [19]. The average NLR was 12.1 and a cut-off of 22.85 was found to be most discriminatory for survival. In addition, Cook et al. evaluated the role of NLR in 100 patients of advanced age, who underwent elective colorectal resection, predominantly for malignant disease [20]. An NLR higher than 9.3 on the first postoperative day was significantly associated with postoperative complications. It is worth mentioning that complications were defined differently in most of the studies, thus comparison of results is difficult. In the present study we used the Calvien-Dindo classification to enable comparison in future.

A few limitations of the study need to be addressed. The study was conducted retrospectively, thus selection bias cannot be ruled out completely. In particular, we could not include all consecutive patients as the NLR and other inflammation parameters were not available in all of them; however, we enrolled a high number of patients, therefore we still believe that the results are of high clinical relevance.

## Conclusion

Peripheral blood derived NLR represents an easily available marker with increased values in CD patients with an intraoperative abscess and an inflammatory mass; however, the clinical role of NLR in predicting 30-day morbidity following surgery for CD is limited. Notably, patients with malignant tumors in resected specimens had significantly higher NLRs, which could be taken into account for clinical decision-making, as these patients will require more extensive intestinal resections. Future studies are necessary to define the role of NLR in CD and malignant diseases.
